# Comparison of the ocular microbiome between chronic Stevens-Johnson syndrome patients and healthy subjects

**DOI:** 10.1038/s41598-020-60794-w

**Published:** 2020-03-09

**Authors:** Thanachaporn Kittipibul, Vilavun Puangsricharern, Tanittha Chatsuwan

**Affiliations:** 1 Excellence Center for Cornea and Limbal Stem Cell Transplantation, Department of Ophthalmology, King Chulalongkorn Memorial Hospital, Bangkok, Thailand; 2 Center of Excellence for Cornea and Stem Cell Transplantation, Faculty of Medicine, Chulalongkorn University, Bangkok, Thailand; 3 Department of Microbiology, Faculty of Medicine, Chulalongkorn University, Bangkok, Thailand; 4 Antimicrobial Resistance and Stewardship Research Unit, Faculty of Medicine, Chulalongkorn University, Bangkok, Thailand

**Keywords:** Microbiome, Medical research

## Abstract

Stevens - Johnson syndrome (SJS) has manifestation through the exfoliation of epidermis and mucosal tissue. Ocular surface is usually affected in acute and chronic stage. The patients are usually suffered from chronic ocular sequelae including symblepharon, limbal stem cell deficiency, etc. Furthermore, ocular microbiome may also be altered in SJS. This is prospective, age and sex matched analytical study which including 20 chronic SJS patients and 20 healthy subjects for specimen collection from inferior conjunctiva for microbiome analysis by conventional cultures and Next-Generation Sequencing (NGS) methods. Significant higher proportion of positive-cultured specimen was demonstrated in SJS group (SJS group 60%, healthy 10%, p-value = 0.001). In addition, NGS which providing high-throughput sequencing has demonstrated the greater diversity of microbial species. The higher proportion of pathogenic microorganisms including *Pseudomonas* spp., *Staphylococcus* spp., *Streptococcus* spp., *Acinetobacter* spp. was shown in SJS group. Ocular surface in SJS is usually occupied by more diverse microorganisms with increased proportion of pathogenic species. This condition may affect chronic inflammation and opportunistic infections in SJS group. In order to prevent and treat infection in these patients, appropriate antibiotics based on bacterial examination should be considered as the first-line treatment in the SJS patients.

## Introduction

Stevens - Johnson syndrome (SJS) is an abnormal immune-mediated responses stimulated by medications such as sulfonamides, allopurinol, cold medicines including paracetamol^1^ and systemic infections caused by virus and mycoplasma. Incidence of SJS is 1.2–6 out of 1,000,000 persons per year^2^. Although the incidence of diseases is low, the mortality rate is high ranging from 1% to 5%^2^. Patients usually present with severe, acute blistering disorders that affect the skin and mucous membranes involving oral mucosa and ocular surface^3^. Ocular involvement has been reported in 25–75% of SJS patients^2^. In the acute phase, patients usually develop conjunctivitis, corneal abrasion, and pseudo-membrane formation. While chronic ocular sequelae develop in 35% of cases, including eyelid irregularity, symblepharon formation, limbal stem cell deficiency (LSCD), and keratinization, leading to poor vision^2^.

Recent interests are focused in the change of ocular microbiomes in this devastating disease. Several studies reported alteration of the ocular microbiomes in SJS patients, compared to healthy subjects. In healthy eyes, the most common microorganisms are *Staphylococcus coagulase negative, Corynebacterium, and Propionibacterium*^4,5^ which are mainly gram-positive bacteria colonized in the ocular surface. In SJS, more pathogenic species are found including gram-negative bacteria. Frizon, *et al*. reported different compositions of microorganisms in SJS patients consisting of 55% of gram-positive cocci, 19% of gram-positive bacilli, and 25% of gram-negative bacilli^4^. They also found that these pathogenic organisms showed higher tendency of antimicrobial resistance pattern to conventional antibiotics. Alterations in the ocular microbial community may link to higher rate of infection in SJS patients who underwent ocular surgeries, such as high incidence of endophthalmitis following keratoprosthesis^6^, ocular surface reconstruction by amniotic membrane with living related corneal limbal/conjunctival allograft^7^. Sotozono, *et al*.^8^ also reported that SJS was a significantly associated factor for patients to develop methicillin-resistant *Staphylococcus aureus* (MRSA)- methicillin-resistant *Staphylococcus epidermidis* (MRSE) keratitis. This altered microbiome may be affected by abnormality of host innate immunity as reported by Ueta and Kinoshita^9^.

Reported studies in ocular microbiomes are varied according to different microbial detection methods. There are mainly two types; (1) microbiologic identification using culture technique and (2) molecular biological method. Conventional culture method usually demonstrates the lower number of microorganisms due to the limitation of detecting slow-growing and uncultivable microorganisms^5^. Molecular biological methods, on the contrary, show higher rates of microbial detection^10,11^. Two commonly-used molecular methods are 16S rRNA gene sequencing by Sangen method and the modern sequencing technology, next-generation sequencing method (NGS). NGS, a high throughput sequencing, has the advantage of large-amount database detection with reduced analytic time^12^. However, there are few reports about ocular microbiome change using NGS method in some other ocular surface diseases, such as dry eyes, contact lens wearing, chronic ocular graft-versus-host disease, etc. And to our knowledge, there is no report using NGS method to identify microbiome changes in SJS subjects. Therefore, we conducted this study using both microbiologic and NGS studies to identify ocular microbiome changes in SJS compared with healthy subjects.

## Results

### Clinical characteristics

A total of 40 conjunctival swabs were collected from 20 eyes of 20 chronic SJS patients and 20 eyes of 20 healthy subjects. We included 10 men (25%) and 30 women (75%) with a mean of age 44.5 in SJS group and 44.2 in healthy group (Table 1). In SJS group, the most common cause was drug-induced. The most common causative drug in our series was penicillin (Table 2). The duration of illness from the onset of SJS ranging from 3 months to 30 years. Chronic SJS patients were classified according to severity score based on chronic ocular complications^13^. The score of all patients ranged from 0–27 and the overall median score was 7 (Table 3).

**Table 1:** Demographic data.

	SJS group (n = 20)	Healthy group (n = 20)
Age (yrs)		
Mean (range)	44.5 (20–77)	44.2 (24–77)
Median (Min, Max)	43 (20, 81)	42 (24, 77)
Male/Female	5/15	5/15

**Table 2:** Etiology of SJS patients.

Etiology of SJS	SJS patients
Anti-epileptic drugs	
Carbamazepine*	2
Phenytoin*	2
Antibiotics	
Penicillin*	6
Cephalosporins	2
Fluoroquinolones	1
Macrolides	1
Ampicillin/Sulbactam	1
Sulfamethoxazole/Trimethoprim	1
Dapsone	1
Others	
NSAIDs	1
Allopurinol	2

*Paracetamol was reported as a combined drug with 3 patients as 1 patient of carbamazepine-allergy group, 1 patient of phenytoin-allergy group and 1 patient of penicillin-allergy group.

**Table 3:** Severity grading score in chronic SJS group.

SJS	Severity grading	SJS	Severity grading
S1	0	S11	26
S2	2	S12	9
S3	0	S13	16
S4	16	S14	7
S5	0	S15	1
S6	13	S16	4
S7	0	S17	0
S8	27	S18	7
S9	10	S19	14
S10	19	S20	3

### Microbiological examination

Among 20 eyes of chronic SJS group, 12 eyes (60%) had positive cultures for bacteria. Predominant microorganisms detected from conventional culture methods were Gram-positive cocci and Gram-positive bacilli. Eleven different kinds of bacteria were identified. Among these, *Corynebacterium* spp. was found in 4 eyes (20%), *Streptococcus pneumoniae* was found in 2 eyes (10%), *Streptococcus agalactiae* was found in 1 eye (5%), *Staphylococcus aureus* was found in 1 eye (5%) (Table 4).

**Table 4:** Results of conventional culture method (culture-positive group).

	SJS group (eyes)	Healthy group (eyes)
No growth	**8 (40.0%)**	**18 (90.0%)**
Growth*	**12 (60.0%)**	**2 (10.0%)**
Gram-positive bacilli	**6**	**0**
*Bacillus amyloliquefaciens*	1	—
*Cellulomonas* species	1	—
*Corynebacterium* species	4	—
Gram-positive cocci	**7**	**2**
*Staphylococcus aureus*	1*	—
*Staphylococcus capitis*	1	—
*Staphylococcus epidermidis*	1	2
*Streptococcus agalactiae*	1	—
*Streptococcus milleri*	1	—
*Streptococcus pneumoniae*	2	—
Gram-negative bacilli	**1**	—
*Proteus mirabilis*	1**	—

*S. aureus showed resistance to benzylpenicillin, ciprofloxacin, moxifloxacin, erythromycin, clindamycin and trimethoprim/sulfamethoxazole.

**P. mirabilis showed resistance to tetracycline, trimethoprim/sulfamethoxazole.

Multiple organisms were isolated from 2 patients (10%) including *Corynebacterium striatum with Staphylococcus capitis* (1/20) and *Streptococcus milleri with Corynebacterium jeikeium* (1/20). Among 20 eyes of healthy group, 2 eyes (10%) had positive culture. The isolates identified was *Staphylococcus epidermidis*. We did not find multiple isolates in healthy group.

The analysis of microbial detection between 2 groups was performed and showed a significantly higher rate of bacterial positivity in chronic SJS group (60%) compared with healthy group (10%) (*p*-value = 0.001, 95% confident interval 24.8–75.2)

Among chronic SJS patients, the severity score affected the results of positive culture. We found the median score of 12 in culture-positive group and the median score of 1 in culture-negative group. Statistical analysis showed a significant difference between the 2 groups with a *p*-value 0.016.

### Antimicrobial susceptibility

In chronic SJS, the highest percentage of antibiotic resistance is shown in *S. aureus* to benzylpenicillin, ciprofloxacin, moxifloxacin, erythromycin, clindamycin and trimethoprim/sulfamethoxazole followed by *P. mirabilis* which showed resistance to tetracycline and trimethoprim/sulfamethoxazole.

In the healthy group, *S. epidermidis* was detected in 2 patients and showed resistance for benzylpenicillin and oxacillin. These two isolates were considered the microorganisms as methicillin-resistant *Staphylococcus epidermidis* (MRSE).

### Next-generation sequencing analysis

Illumina sequencing of 16S rRNA genes generated quality filtered reads of 2,838,365 from all specimens. A total of 40 specimens were rarefied. Rarefaction curves derived from the observed OTU number which were flattened around 20,000 reads.

### Core microbiome

To identify core microbiome by 100% of core OTUs samples matching, we found Pseudoalteromonadaceae, Vibrionaceae, Burkholderiaceae, Enterobateriaceae in SJS patients while Pseudoalteromonadaceae and Vibrionaceae were identified as core microbiome in healthy subjects.

### Taxonomic composition of microbial community on ocular surface

**Figure 1:**
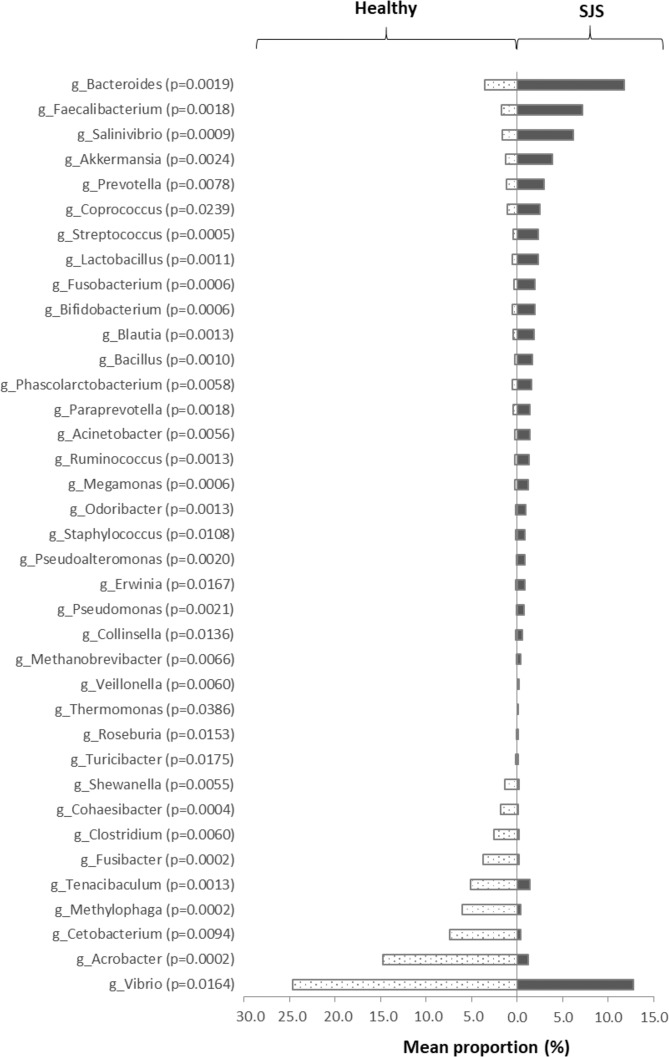
Significant difference of taxa abundance. This figure represents the significant difference of taxonomic level of each genus comparing between SJS patients and healthy subjects. We analyzed the proportion of each genus by Mann-Whitney U test. The p-value less than 0.05 was defined as statistically significant difference.

We identified the taxonomic composition of ocular surface microbiome by 16S rRNA sequencing at phylum and genus level. In SJS group, 15 bacterial phyla were identified. Predominant phyla consisted of Proteobacteria (34.80%), Firmicutes (23.80%), Bacteroidetes (13.10%), Tenericutes (11.9%), Actinobacteria (9.8%) and etc. The significant difference of relative abundance between 2 groups was shown *Acrobacter, Streptococcus, Lactobacillus, Bacillus, Bifidobacterium, Bacteroides, Pseudomonas, Acinetobacter, Staphylococcus, Pseudoalteromonas, Clostridium*, etc. Data was analyzed by Mann-Whitney U test. A *p*-value less than 0.05 was considered as having a statistically significant difference. (Fig. 1) When comparing with healthy subjects, the relative proportion of *Lactobacillus, Bacteroides, Pseudomonas, Staphylococcus, Streptococcus, Bacillus, Acinetobacter* were higher in SJS group.

### The alpha-diversity

**Figure 2:**
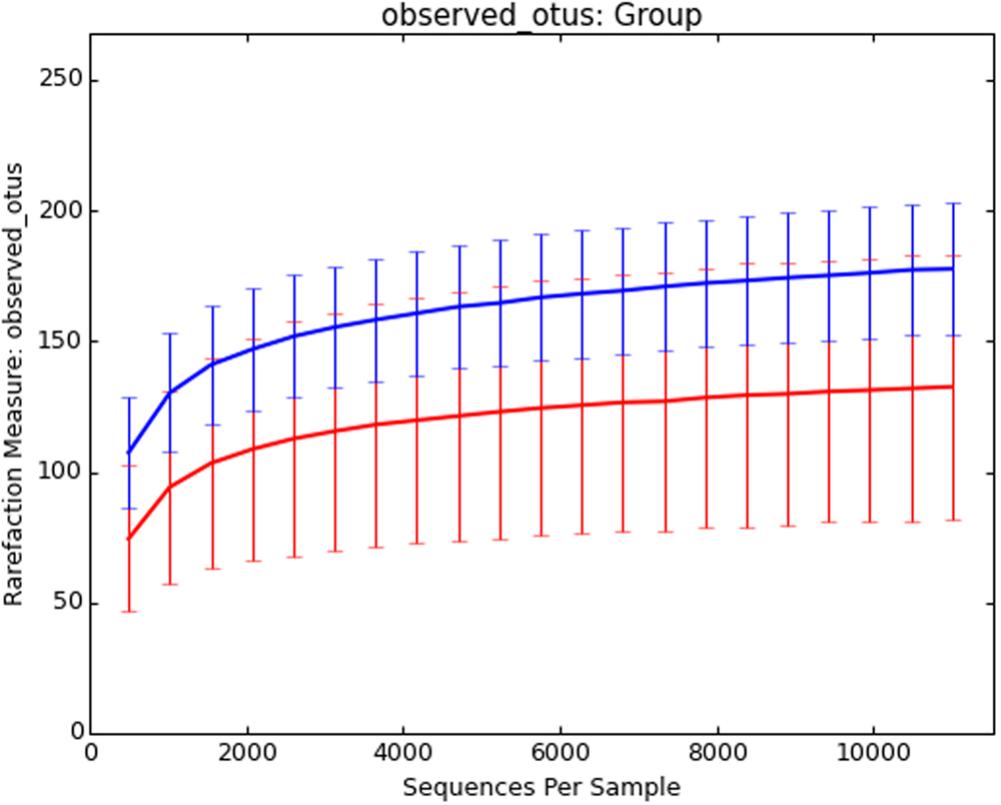
Rarefaction curves. This figure demonstrates the number of OTUs which calculated from individual samples. This curve shows the plots of the number of species in SJS (blue line) and healthy groups (red line). Greater amount of OTUs were observed in SJS. group.

**Figure 3:**
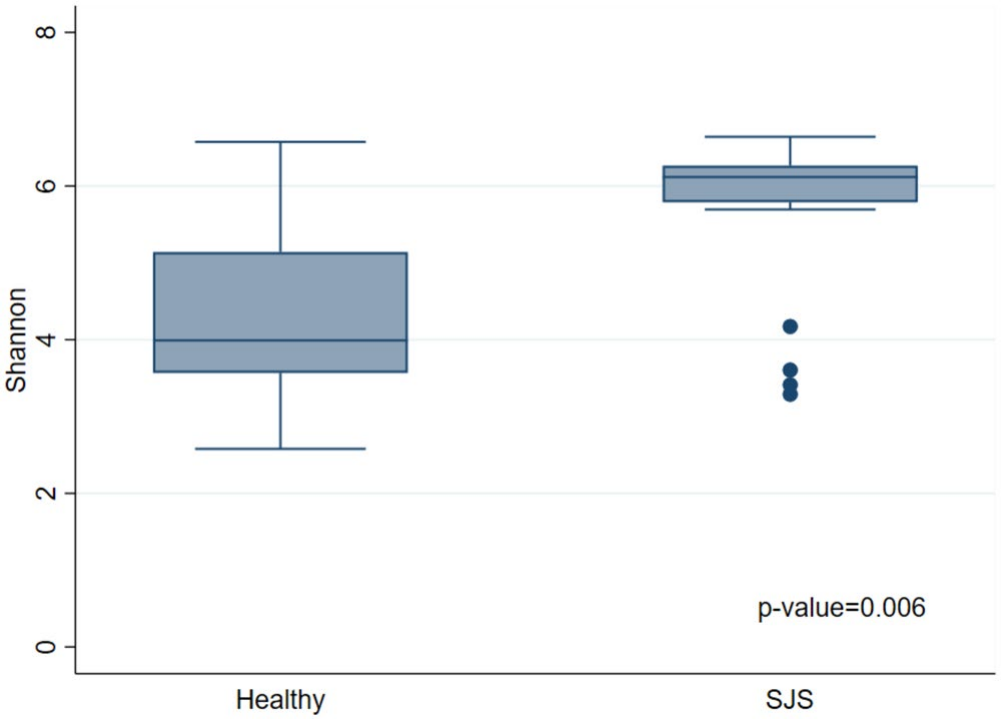
Shannon Index. This figure shows the difference of microbial community between healthy and SJS. Significant difference is observed in Shannon diversity index with p-value = 0.006.

**Figure 4:**
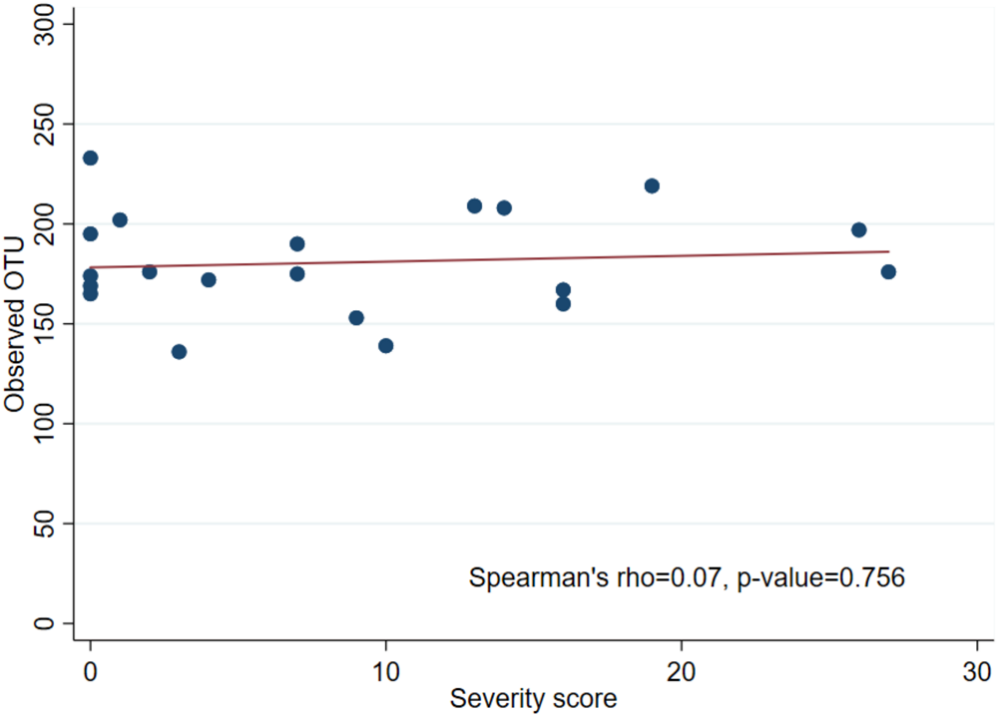
Spearman’s correlation. This figure shows the correlation between the numbers of OTUs and the severity score of SJS patients. X-axis shows the numbers of OTUs detected from NGS. Y-axis shows the severity grading score by Sotozono, *et al*. There was no correlation between the numbers of OTUs and severity score in our study (Spearman’s rho = 0.07, p-value = 0.756).

We used rarefaction curve which is derived from observed OTU number to assess the species richness in each single specimen. The rarefaction curve has approached asymptotes. The higher diversity was demonstrated in SJS patients. (Fig. 2). To analyze the species richness by diversity index, Shannon index showed a statistically significantly higher abundance in SJS patients (*p*-value 0.0063) (Fig. 3). We also performed Spearman’s correlation analysis to see whether the severity score could affect the number of OTUs (Fig. 4). However, we did not find correlation between severity score and the number of OTUs in our series.

### The beta-diversity

**Figure 5:**
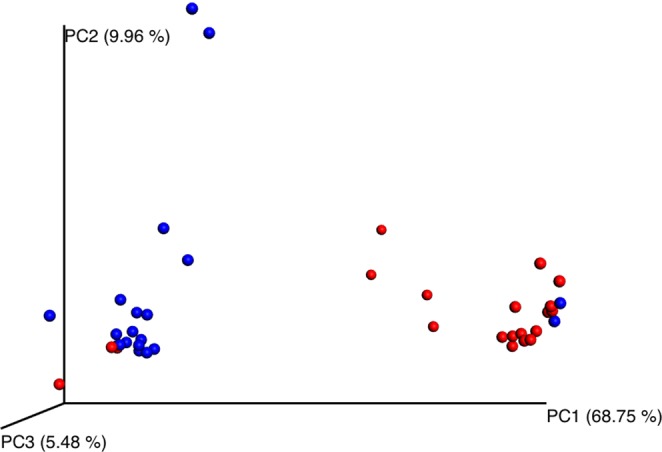
Principal Coordinate Analysis (PCoA). This figure shows beta diversity in conjunctival samples between SJS patients and healthy subjects. Weighted UniFrac distances were used to evaluate diversity between groups. PCoA plots show the clusters of ocular microbiota in SJS (blue) and healthy (red) groups. Adonis test statistic showed significant difference with p-value = 0.001 and R^2^ = 0.32 using weight unifrac distance matrix.

To determine differences between the 2 groups, we applied 3D principal coordinate analysis (PCoA) by using weighted UniFrac distances to represent scale of difference between groups. The significant dissimilarity between 2 groups is demonstrated by the definite separation of 2 clusters on PCoA plots. (Fig. 5)

## Discussion

Knowledge in the human microbiome has been discovered and reported, using complex analysis of genome technology. The human microbiome is believed to maintain equilibrium of the specific organ which it has colonized. A previous study from Wikoff *et al*.^14^ reported the association between microbiota and gut metabolism in mammalian model. On the ocular surface, alterations of microbiome were demonstrated in several conditions such as aging change^15^, geographic location, nutritional status, contact lens wear^16^, dry eye syndrome^17^, allergy, chronic inflammation^18^ and Sjögren Syndrome^19^. However, most of the studies used conventional culture method while few used the molecular method. At present, there is still limited information about the ocular microbiome which is analyzed by molecular method. To our knowledge, there is no study that compares the ocular microbiome between SJS and healthy groups. Our study aimed to report the ocular microbiome in SJS and healthy by using conventional culture method and NGS analysis.

In this study, the result from conventional culture method showed *Corynebacterium* spp. and *Streptococcus* spp. as prominent species in SJS patients which was similar to previous studies. Venugopal, *et al*.^20^ from India reported *Corynebacterium* spp. as the prominent species in SJS patients. Frizon, *et al*.^21^ also found coagulase-negative staphylococci and *Corynebacterium* spp. as the prominent species in SJS group. We also found a higher incidence of culture-positive specimens in SJS group comparing with healthy subjects (*p* = 0.001) as has been shown in the study from Venugopal, *et al*. A greater variation of bacterial species was found in SJS group which is consistent with previous studies^20^.

Comparing the severity scores in chronic SJS group, significantly higher score was shown in culture-positive SJS groups (*p*-value = 0.016). According to the severity score of chronic ocular SJS proposed by Sotozono, *et al*.^13^, they reported the correlation between grading score and visual outcome. In our study, we found the association between high grading score and higher rate of culture-positive specimens. Another interesting finding is the presence of *S. agalactiae* in one patient who had a high severity score of 26. Since *S. agalactiae* resides as commensal bacteria of gastrointestinal and genitourinary tract, the presence of this organism on the ocular surface is considered to be atypical colonization. These results imply that the higher severity score may affect greater changes of ocular surface microbiome with higher possibility to detect atypical bacterial species. We did not find multidrug resistance strains (i.e.methicillin-resistant *Staphylococcus aureus* or MRSA) which was different from previous studies from India and Japan, which reported high rate of antibiotic resistance in viridans streptococci^20^ and significant high incidence of methicillin-resistant *Staphylococcus* infection^8^. The variation of antibiotic-resistant patterns in each country may be related to different bacterial distribution, patterns of antimicrobial use and antimicrobial resistance patterns in each country^22^.

Analyses of core microbiome of SJS and healthy groups showed Pseudoalteromonadaceae and Vibrionaceae as 100% of core OTUs in both groups. Besides these families, we also found Burkholderiaceae and Enterobacteriaceae as 100% of the core OTUs in SJS group. The difference of core microbiome may be another explanation for severe atypical infection in SJS patients. In addition, the analysis of bacterial species revealed an increased proportion of *Acinetobacter* spp*., Bacteroides* spp.*, Corynebacterium* spp.*, Pseudomonas* spp.*, Staphylococcus* spp.*, Streptococcus* spp., etc in chronic SJS patients, whereas the healthy group demonstrated higher proportions of *Acrobacter* spp.*, Clostridium* spp*., Fusibacter* spp., etc. Gram-positive bacteria was found to dominate the ocular surface when detected by culture method. In contrast, 16S rRNA gene sequencing showed higher diversity of bacterial species and revealed that the predominant bacteria was gram-negative bacteria (i.e. Proteobacteria). The more extensive species in NGS analysis may indicate the better performance to detect small amounts of microbes, slow-growing and non-cultivable microbial species^23^.

Frizon, *et al*. reported the increase of bacterial diversity in SJS group^21^. In this study, several analyses were performed to compare the α-diversity in SJS and healthy groups. We created the rarefaction curve and found a higher proportion of observed OTUs in SJS patients comparing with healthy patients. In addition, the Shannon index showed a significantly higher diversity in SJS patients compared to healthy group (*p*-value 0.0063). These analyses indicated higher species abundance in SJS group. To consider about the effect of severity score in SJS group on the number of OTUs, we performed Spearman’s correlation analysis and did not find the correlation between severity score and the number of OTUs. To compare the differences of microbial communities, we performed the PCoA. This analysis showed that most data were clustered into 2 groups. Further assessment using UniFrac based distance demonstrated the tendency of difference in microbial community between SJS and healthy groups.

Our findings demonstrated the alteration of ocular microbiome in term of diversity and higher proportion of potentially pathogenic microorganism. These changes may be related to severe ocular surface abnormalities including epithelial damage, reduction of aqueous and mucin layer, etc., as reported by Shimizu, *et al*.^23^. The dysfunction may impair the ability to eliminate pathogens from ocular surface and affect microbial community. In addition, Ueta and Kinoshita^9^ reported the abnormality of innate immune response in SJS which may affect balance between mucosal immunity and pathogenicity of bacteria. This altered immunity may be another factor that impacts on ocular surface microbiome.

Furthermore, the remarkable change of ocular surface microbiome in chronic SJS patients may be an important cause of chronic-recurrent ocular surface inflammation^24^. Therefore, severe ocular infections which usually presented in SJS patients, may be the result of the microbiome changes. In order to prevent and control infection in SJS patients, clinicians should strongly consider appropriate antibiotics for these patients.

The limitations of this study are small sample size and because that some information may not be able to display the significant difference between groups. Although the data was mainly derived from Thai people, it may be applicable for Asian population especially in Southeast Asia. To analyze other variables that may affect ocular microbiome (i.e. the duration of SJS), further study in a larger group should be conducted.

## Conclusion

In summary, the ocular surface microbiome of SJS and healthy subjects was clearly demonstrated by NGS analysis in this study. We found a higher diversity of microbiome with an increased proportion of some opportunistic pathogens in SJS group. In addition, significantly higher rates of culture-positive specimens were demonstrated in SJS group. The association between culture-positive specimens and the severe grading score of chronic SJS was also demonstrated. Further studies should be pursued to identify whether or not these alterations may be the underlying cause of persistent inflammation and recurrent infections which lead to worsened visual prognosis in SJS patients.

## Material and Methods

### Ethical approval and clinical trial registration

The study adhered to the tenets of the Declaration of Helsinki and was approved by the Institutional Review Board, Faculty of Medicine, Chulalongkorn University with IRB No. 399/61 and COA No 735/2018. The study was approved by Thai Clinical Trial Registration with TCTR No. TCTR20190505001.

### Subject recruitment

The study was conducted on 20 chronic SJS patients (onset of SJS more than 4 weeks) and 20 healthy subjects who were recruited from the outpatient department of Department of Ophthalmology, King Chulalongkorn Memorial Hospital. Inclusion was carried out in subjects aged over 18 years old. Exclusion criteria were use of topical antibiotics in the past 4 weeks, use of contact lens or punctal plugs for the same period, history of ocular and periocular infection within 4 weeks and a history of previous ocular surgery in past 3 months. Patients’ clinical history were documented. Complete eye examinations were done by T.K. In chronic SJS patients, severity grading score of ocular complications was classifies as corneal (i.e. superficial punctate keratopathy, epithelial defect, loss of the palisades of Vogt), conjunctival (hyperemia, symblepharon formation), and eyelid (trichiasis, mucocutaneous junction involvement, meibomian gland involvement) complications^13^. This score was evaluated and graded on a scale from 0–3 in each components according to the severity. Total score was ranged from 0 to 39. Healthy subjects were defined as subjects who had no problems of chronic ocular allergy, inflammation and any other ocular surface diseases. Healthy subjects were selected randomly from the outpatient department by matching with age and sex of SJS group. Informed consents were obtained from all patients at the beginning of the study.

### Specimen collection

Conjunctival swabs were performed in 20 eyes of 20 SJS patients, 20 eyes of 20 healthy subjects. In healthy group, the eyes included into the study were selected by the subjects. In chronic SJS group, the worse eyes were selected into the study. After instilling topical anesthesia (0.5% Tetracaine Hydrochloride Solution, Alcon®) for 3 minutes, sterile cotton swabs were applied from the medial to the lateral part of inferior fornix of conjunctival sac without touching eyelids 3 times. If the symblepharon presented, the investigator would avoid swabbing in that area. After completing, the swabs were placed in sterile transport media.

### Culture and antibiotic sensitivity

After placing conjunctival swab into transport media for 10 minutes, the dissolved media was then collected by a sterile dropper. This specimen was later placed on chocolate agar plate. The plates were incubated in a CO_2_ incubator at 37 °C, 5% CO_2_ and examined daily for any growth for a week. Bacterial identification was determined by biochemical tests and the API system.

The identified microbial isolates were subjected to antimicrobial susceptibility testing for benzylpenicillin, ampicillin, amoxicillin/clavulanic acid, piperacillin/tazobactam, cefazolin, cefuroxime, ceftriaxone, oxacillin, ertapenem, imipenem, meropenem, amikacin, gentamycin, ofloxacin, ciprofloxacin, moxifloxacin, erythromycin, clindamycin, linezolid, teicoplanin, vancomycin, tetracycline, rifampicin and trimethoprim/sulfamethoxazole using the Kirby–Bauer disk diffusion method and the Vitek2 system. The results were interpreted according to CLSI guideline.

### Next-generation sequencing analysis

16S rRNA gene was amplified. 341F and 805R primers were applied, targeting V3-V4 variable regions and 2X KAPA hot-start ready mix. The amplified condition consisted of an initial denaturation step for 3 minutes at 94 °C, followed by 25 cycles of 98 °C for 20 s., 55 °C for 30 s., and 72 °C for 30 s., followed by a single step final extension step at 72 °C for 5 minutes. Subsequently, 16S amplicon were purified by AMPure XP beads and indexed by 5 µl of each Nextera XT index primer in a 50 µl PCR reaction, followed by 8–10 cycles of PCR condition above. After cleaning of PCR product was completed, the final PCR product was pooled and diluted to final loading concentration at 4 pM. Cluster generation and 250-bp paired-end read sequencing were performed on an Illumina MiSeq at Omics Sciences and Bioinformatics Center (Chulalongkorn University, Bangkok, Thailand).

Sequencing reads quality was examined by FASTQC software. Overlapping paired-end reads were assembled using PEAR. FASTX-Toolkit filtered out assembled reads that do not have a quality score of 30 at least 90% of bases, and removed the reads that are less than 400 bp long. Chimeras were removed by the UCHIME method^25^ as implemented in vsearch1.1.1^26^ using uchime_ref option against chimera-free Gold RDP database. OTU picking was performed with the *pick_open_reference_otus.py* command in QIIME 1.9.0, specifying that we used SortMeRNA for reference picking, and taxonomic assignments were conducted against Greengenes 97% database. Subsequently, the subsampled failure reads were clustered de novo using SUMACLUST. After the OTU picking, we filtered OTUs that were supported by less than 0.1% reads out of the analysis. To confirm the even sequencing depth across samples, 21,060 of sequences per sample were randomly subsampled for analysis of bacterial communities. Then, alpha diversity estimates were computed for phylogenetic diversity (PD), chao1, observed OTUs, Shannon diversity and a rarefaction curve were generated. In addition, beta diversity was analyzed by computing weighted UniFrac distances between samples to create principal coordinate analyses (PCoA).

### Statistical analysis

Demographic data including age, sex were analyzed using descriptive methods. Chi-square test was used for statistical analysis in comparison of positive-cultured specimens between SJS and healthy groups. Mann-Whitney U test was performed to analyze the difference of severity score between positive and negative cultured groups. Spearman’s correlation was performed to analyze the correlation between SJS severity score and the number of OTUs in each patient. The difference of species abundance was analyzed by Mann-Whitney U test. P-values less than 0.05 were considered statistically significant and Cohen’s d effect size was calculated and presented.

## Data Availability

The data which analyzed from the clinical isolates that support the results of this study are available upon reasonable request from the corresponding author VP.

## References

[1] Jongkhajornpong P (2018). Association between HLA-B*44:03-HLA-C*07:01 haplotype and cold medicine-related Stevens-Johnson syndrome with severe ocular complications in Thailand. Br. J. Ophthalmol..

[2] Jain R (2016). Stevens-Johnson syndrome: the role of an ophthalmologist. Surv. Ophthalmol..

[3] Mannis, M. *et al*. Erythema Multiforme, Stevens–Johnson Syndrome, and Toxic Epidermal Necrolysis, 4^th^ edition [Mannis, M. (ed.)] Cornea. **50**, 1378–1414. (Elsevier, 2017).

[4] Moeller C (2005). Evaluation of normal ocular bacterial flora with two different culture media. Can. J. Ophthalmol..

[5] Dong Q (2011). Diversity of bacteria at healthy human conjunctiva. Invest. Ophthalmol. Vis. Sci..

[6] Nouri M (2001). Endophthalmitis after keratoprosthesis. Arch. Ophthalmol..

[7] Gomes J (2003). Amniotic membrane with living related corneal limbal/conjunctival allograft for ocular surface reconstruction in Stevens-Johnson syndrome. Arch. Ophthalmol..

[8] Sotozono C (2002). Methicillin-resistant Staphylococcus aureus and methicillin-resistant Staphylococcus epidermidis infections in the cornea. Cornea..

[9] Ueta M, Kinoshita S (2012). Ocular surface inflammation is regulated by innate Immunity. Prog. Retin. Eye Res..

[10] Egushi H (2017). Diagnostic approach to ocular infections using various techniques from conventional culture to next-generation sequencing analysis. Cornea..

[11] Huang Y, Yang B, Li W (2016). Defining the normal core microbiome of conjunctival microbial communities. Clin. Microbiol. Infect..

[12] Kasai, C. *et al*. Comparison of the gut microbiota composition between obese and non-obese individuals in a Japanese population, as analyzed by terminal restriction fragment length polymorphism and next-generation sequencing. *BMC Gastroenterol*. **15**, 10.1186/s12876-015-0330-2 (2015). 10.1186/s12876-015-0330-2 PMC4531509 26261039

[13] Sotozono C (2007). New grading system for the evaluation of chronic ocular manifestations in patients with Stevens–Johnson syndrome. Ophthalmology..

[14] Wikoff WR (2009). Metabolomics analysis reveals large effects of gut microflora on mammalian blood metabolites. Proc. Natl Acad. Sci..

[15] Wen X (2017). The influence of age and sex on ocular surface microbiota in healthy adults. Invest. Ophthalmol. Vis. Sci..

[16] Shin, H. *et al*. Changes in the Eye Microbiota Associated with Contact Lens Wearing. *mBio*. **7**, 10.1128/mBio.00198-16 (2016). 10.1128/mBio.00198-16 PMC4817251 27006462

[17] Graham J (2007). Ocular pathogen or commensal: a PCR-based study of surface bacterial flora in normal and dry eyes. Invest. Ophthalmol. Vis. Sci..

[18] Lee S, Oh D, Jung J, Kim J, Jeon C (2012). Comparative ocular microbial communities in humans with and without blepharitis. Invest. Ophthalmol. Vis. Sci..

[19] De Paiva, C. *et al*. Altered mucosal microbiome diversity and disease severity in Sjögren syndrome. *Sci. Rep*. **6**, 10.1038/srep23561 (2016). 10.1038/srep23561 PMC4834578 27087247

[20] Venugopal R (2016). Conjunctival microbial flora in ocular Stevens–Johnson syndrome sequelae patients at a tertiary eye care center. Cornea..

[21] Frizon L (2014). Evaluation of conjunctival bacterial flora in patients with Stevens-Johnson syndrome. Clinics..

[22] Khor W (2018). The Asia cornea society infectious keratitis study: a prospective multicenter study of infectious keratitis in Asia. Am. J. Ophthalmol..

[23] Shimizu E (2019). Commensal microflora in human conjunctiva; characteristics of microflora in the patients with chronic ocular graft-versus-host disease. Ocul. Surf..

[24] Ueta M (2008). Innate immunity of the ocular surface and ocular surface inflammatory disorders. Cornea..

[25] Edgar R, Haas B, Clemente J, Quince C, Knight R (2011). UCHIME improves sensitivity and speed of chimera detection. Bioinformatics..

[26] Rognes T, Flouri T, Nichols B, Quince C, Mahé F (2016). VSEARCH: a versatile open source tool for metagenomics. PeerJ..

